# Maternal Nursing

**Published:** 1907-11-30

**Authors:** 


### Gynæcology.

### MATERNAL NURSING.

A definite number of instances, in which the mother is unable to nurse her baby, are found due on investigation to the neglect of simple precautions in the management of the breasts and nipple during pregnancy and lactation. Much of this neglect arises from ignorance and can be guarded against by a little simple advice from the family doctor.

In a primipara no special attention is needed for the breasts in most cases. But in those with exceptionally large breasts and in multiparæ a moderate degree of support by a suitably arranged handkerchief or bandage will relieve the dragging sensation, due to the increasing size and weight. The clothes should be loosely arranged to prevent pressure. The skin in the sub-mammary folds should be washed night and morning and freely dusted with a drying powder, such as zinc oxide and starch.

### How to Prevent Sore Nipples.

Attention to the nipples is of even greater importance, for they are liable to excoriation and fissure during suckling, and to secondary infection which may give rise to mastitis or mammary abscess. During the last three months of pregnancy the nipples should be washed daily, and freely anointed with benzoated lard, cacao-butter, vaseline, olive oil, or lanoline. If they are small or retracted, the expectant mother must be taught to use the finger and thumb to draw them out.

Treatment of this nature gives them a proper size and shape, and makes the epithelial covering soft and pliable. Neither direct nor indirect suction should be used to draw out the nipples. Neither astringent nor spirituous lotions should be applied, for they harden the skin and make it more liable to crack. For protection during the last few months of pregnancy, if the nipples are swollen and prominent, a pad of antiseptic wool is sufficient. It should be frequently changed, if there is much overflow of milk. It is from neglect of these simple precautions that the painful fissure of the nipple so often arises during lactation and compels the mother to give up nursing.

After the birth of the child want of cleanliness, constant moisture from galactorrhœa, or too frequent suckling, may cause excoriation or abrasion of the epithelium. Infection of the raw surface and the mechanical effects of suckling prevent healing, and the fissure develops. Apart from the pain to the mother and the liability to mammary infection, a fissure may be injurious to the child in consequence of the amount of blood swallowed.

### Some Further Points in Treatment.

It is obvious that the main treatment is essentially preventive. During lactation the nipples should be bathed with cold water before and after nursing. Olive oil, etc., should be applied two or three times a day. If there is the least excoriation, it should be treated at once with zinc ointment; balsam of Peru one drachm, with half an ounce each of cold cream and lanoline; or, if an astringent is needed, tannic acid, gr. xv, and Friar's balsam, half a drachm, to the ounce of vaseline. A simple measure is to paint on the nipples a protective coating of raw white of egg before and after nursing, washing well first.

More often attention is not drawn to the nipples until an actual fissure has formed. The fissured nipple should be dried with antiseptic wool and painted freely with a mucilaginous solution of boric acid, gr. x to the ounce, after each nursing, the parts being washed with warm water and painted with raw white of egg before the child is put to the breast. Should this fail, the fissure may be touched every other day with a finely pointed stick of silver nitrate. A more thorough method consists in washing with 5 per cent. boric acid solution, anæsthetising with cocaine, painting with a 10 per cent. solution of silver nitrate, drying with absorbent wool, and finally painting with egg albumin. During the treatment the mother should continue nursing with the aid of the nipple shield.

A 5 to 10 per cent. solution of cocaine, applied half an hour before nursing, will relieve pain, but the nipple must always be carefully washed before allowing the child to suck.

Sometimes all these methods fail and the breast-feeding will have to be temporarily omitted, the child being fed alternately from the unaffected breast and on an artificial milk mixture. The activity of the gland can often be successfully maintained by the systematic use of the breast pump.

### Mammary Abscess.

Lymphangitis and abscess of the breast are still more serious affections, and generally arise through infection of the nipple in the manner already stated. Possibly abscess can be induced by inefficient emptying of the breast during the first few days after confinement, provided an infective organism makes its way into the congested gland through the blood, lymph, or ducts of the nipple. Suckling and the use of the breast-pump must be prohibited, if there is an abscess or even mastitis. Give the mother a saline cathartic, such as a full dose of Epsom salts, two-grain doses of quinine three or four times a day, and apply equal parts of extract of belladonna and glycerine or lead lotion locally. If there is pus, open and drain the abscess. In some cases, treated at the onset, the inflammatory process subsides, milk secretion is continued, and nursing can be resumed.

Tuberculous and malignant disease of the breast are both uncommon in nursing women, and are obvious bars to suckling. Evidence of old mastitis is not a contra-indication, but the child must be carefully watched and weighed regularly, for mastitis interferes with the full activity of the affected gland, and the child may get an insufficient supply of food.

